# In Situ Catalytic Modification of Phenolic Resin Pyrolytic Carbon Using Cupric Tartrate-Derived Cu Nanoparticles: Microstructure Evolution and Oxidation Behavior

**DOI:** 10.3390/ma19132821

**Published:** 2026-07-02

**Authors:** Pengcheng Jiang, Huidong Tang, Xin Xiong, Zhi Wu, Wei Zhang, Wenting Wang, Jingdan Yan, Yao Luo, Yong Su, Siqi Zhu, Can Xia, Ziyue Huang, Yue Gong, Zhoufu Wang

**Affiliations:** 1 School of Materials Science and Engineering, Hunan Institute of Technology, Hengyang 421002, China; 2 Shanxi New Fashion Furnace Industry Group Co., Ltd., Taiyuan 030100, China; 3 The State Key Laboratory of Refractories and Metallurgy, Wuhan University of Science & Technology, Wuhan 430081, China

**Keywords:** cupric tartrate, Cu nanoparticles, carbon nanofiber, oxidation resistance, oxidation activation energy

## Abstract

Phenolic resin is widely used as a binder in high-temperature industries; however, its pyrolysis generally yields isotropic glassy carbon, which strongly influences its high-temperature oxidation behavior. In this work, cupric tartrate was introduced as a catalyst precursor to investigate its effects on the thermal decomposition behavior, microstructural evolution, and oxidation behavior of the phenolic resin pyrolytic carbon. Upon heating, cupric tartrate decomposed at 250–320 °C into nanoscale Cu/Cu_2_O composites, which were then converted into metallic Cu nanoparticles through reduction by gaseous products generated during the pyrolysis of phenolic resin. The in situ formed Cu nanoparticles were associated with the growth of tapered carbon nanofibers (CNFs), reaching maximum lengths of 30–50 μm at 700 °C. Based on the observed microstructural features and established literature reports, a dissolution–precipitation pathway is proposed to rationalize the formation of these CNFs. The presence of Cu-catalyzed CNFs correlates with enhanced structural ordering of the pyrolytic carbon, as reflected by reduced I_D_/I_G_ ratios, and with an increased apparent oxidation activation energy in the selected fitting region (from 103.73 to 137.45 kJ/mol). Overall, this work demonstrates a facile strategy in which cupric tartrate serves as an effective catalyst precursor that generates Cu nanoparticles in situ; these nanoparticles then catalyze CNF growth from phenolic resin, enabling the construction of low-dimensional carbon nanostructures.

## 1. Introduction

Phenolic resin is commonly employed as a binder in high-temperature industrial applications, including carbon-containing refractories [1], ceramics [2], and carbon/silicon carbide composites [3], mainly because it combines a high carbon yield, excellent thermal stability, and appropriate room-temperature rheological properties [4,5,6]. Upon curing, its three-dimensional cross-linked network structure provides a continuous carbon framework after pyrolysis, which is essential for maintaining the mechanical integrity of the resulting carbon materials at elevated temperatures [7,8,9].

However, the thermal decomposition of phenolic resin inevitably generates pores and defects [10]. During pyrolysis between 200 °C and 750 °C, cleavage of methylene and ether linkages releases low-molecular-weight gases, including H_2_O, CO, CO_2_, CH_4_, and H_2_, consequently yielding isotropic glassy carbon with a limited degree of structural ordering [11]. The amorphous carbon framework compromises the mechanical strength of the material and weakens its resistance to oxidation, consequently shortening its service life under oxidizing conditions [12].

To overcome these drawbacks, catalytic structural ordering of phenolic resin using transition metals has attracted considerable attention [13]. Metals such as Fe [14], Co [15], and Ni [16] have been shown to facilitate the conversion of amorphous carbon into highly ordered graphitic domains while also promoting the formation of carbon nanotubes (CNTs) or carbon nanofibers (CNFs) during pyrolysis [17]. These nanostructures can fill pores and enhance structural ordering, thereby improving oxidation resistance. However, catalytic pyrolysis processes involving Fe, Co, and Ni typically require temperatures of 800 °C or higher to achieve effective structural ordering and CNF/CNT growth. Below this temperature, phenolic resin has already undergone most of its structural evolution and gas release, limiting the opportunity for in situ catalytic modification during the critical degradation stage.

Copper has attracted increasing attention as an alternative catalyst because it can promote the growth of CNFs or CNTs at substantially lower temperatures (e.g., 250–500 °C) via chemical vapor deposition or pyrolysis of carbonaceous gases [18]. Several studies have demonstrated that Cu nanoparticles are active for cracking hydrocarbon gases (CH_4_, CO, C_2_H_2_) into carbon nanofibers [19]. Nevertheless, the catalytic performance of Cu is strongly dependent on the particle size, morphology, and spatial distribution of the active metallic species, which are largely determined by the choice of catalyst precursor [20]. Zhang et al. [21] investigated the influence of nano-Cu particle size on the morphology of carbon fibers and found that, under identical reaction conditions, smaller Cu nanoparticles tended to promote the formation of coiled carbon nanofibers, whereas larger Cu particles favored the formation of straight carbon nanofibers. Similarly, Acauan et al. [22] treated bulk copper substrates with a strong alkaline solution and subsequently synthesized carbon nanomaterials (CNMs) on the copper surface via chemical vapor deposition (CVD); they observed that the morphology of the deposited carbon varied with the heat-treatment temperature, indicating that temperature is another critical factor governing Cu-catalyzed carbon synthesis. In a related study, Bhaduri et al. [23] examined the catalytic role of nano-Cu particles in ethylene polymerization and found that the polymerization reaction occurred on the surface of the Cu nanoparticles, and that only Cu particles on the nanoscale exhibited appreciable catalytic activity. Moreover, after heat treatment at 700 °C, the nano-Cu species became embedded within carbon nanofibers and graphite, further confirming that the size of the Cu particles derived from the thermal decomposition of the catalyst precursor is crucial for catalytic performance.

Different copper precursors exhibit distinct decomposition pathways and yield Cu, CuO or Cu_2_O particles with varying sizes and shapes. In addition to particle size, the choice of copper precursor also plays a decisive role. Shaikjee et al. [24] used Cu-based catalysts to synthesize low-dimensional carbon nanostructures (helical, long straight, and amorphous) via CVD and demonstrated that Cu particles derived from different precursors—namely Cu(NO_3_)_2_, CuCl_2_, and Cu(acac)_2_—exhibited distinct morphologies and, consequently, different yields of carbon nanofibers. Importantly, they also noted that the CuO species formed by thermal decomposition of the copper salt precursors were catalytically inactive; only after they were reduced to metallic Cu did catalytic activity emerge. This finding highlights the importance of selecting a catalyst precursor that can yield elemental Cu directly upon thermal decomposition, thereby avoiding the need for a separate reduction step.

In this context, Zhang et al. [25] demonstrated that cupric tartrate can serve as an effective precursor for the synthesis of carbon fiber flower-like networks via catalytic CVD at 300 °C. Upon heating in air to 300 °C, cupric tartrate was found to oxidize to Cu_2_O (34 nm) and CuO (21 nm); subsequent introduction of H_2_ reduced these oxides to Cu nanoparticles (approximately 50 nm). This oxidation–reduction pretreatment highlights the potential of nanoscale copper salts as catalyst precursors. However, the decomposition products and their sizes obtained from the direct thermal decomposition of cupric tartrate under an inert atmosphere remain unclear, and whether such products can catalytically convert the gaseous species evolved during phenolic resin pyrolysis into carbon nanofibers has yet to be explored. Therefore, the present study aims to address these gaps by investigating the thermal decomposition behavior of cupric tartrate under inert conditions and its catalytic effect on the in situ growth of carbon nanofibers from phenolic resin-derived pyrolysis gases.

In this work, cupric tartrate was selected as the catalyst precursor. The effects of cupric tartrate on the pyrolysis behavior, microstructure evolution, and oxidation resistance of phenolic resin-derived carbon are systematically investigated, and the possible catalytic pathway for CNF formation is discussed. This work provides a facile strategy to fabricate high-performance phenolic resin-based carbon materials with improved thermal stability and oxidation resistance.

## 2. Experimental Section

### 2.1. Raw Materials

Phenolic resin with a carbon yield of no less than 55% was employed as the starting material and obtained from Lifa Co., Ltd. (Wuhan, China). Cupric tartrate (C_4_H_4_CuO_6_·xH_2_O, CP, ≥98.5%) was selected as the catalyst precursor and purchased from Aladdin (Shanghai, China).

### 2.2. Sample Preparation

Cupric tartrate was incorporated into the phenolic resin at a loading of 1.0 wt% relative to the resin and thoroughly homogenized. In this work, 1.0 wt% cupric tartrate was selected as the representative loading based on previous Cu-catalyzed carbon-formation studies [26] and on preliminary optimization experiments. A low catalyst addition provides insufficient Cu nanoparticles to effectively catalyze CNF formation, whereas an excessive addition leads to severe agglomeration of Cu particles, which reduces catalytic efficiency and suppresses CNF growth. After curing at 180 °C for 24 h, the sample incorporating cupric tartrate was denoted as PRCu, whereas the phenolic resin without catalyst was referred to as PR. The cured samples were then pyrolyzed in a tube furnace under argon flow (≥99.99%, 100 mL/min), with a heating rate of 5 °C/min to target temperatures of 500, 600, 700, and 800 °C and a holding time of 3 h, respectively. After furnace cooling, phenolic resin-derived pyrolytic carbon (PRPC) was obtained.

### 2.3. Testing and Characterization

The thermal decomposition behavior of the cupric tartrate precursor and its influence on phenolic resin pyrolysis under an argon atmosphere (10 °C/min, 30 °C to 1000 °C) were characterized using a thermogravimetry-differential scanning calorimetry analyzer (TG-DSC, STA449/6/G, Netzsch, Selb, Germany). The oxidation resistance of PRPC was characterized by TG-DSC in air (10 °C/min, 30 °C to 1000 °C). The oxidation process of the phenolic resin carbonization product mainly consists of three steps: (1) Oxygen in the air adsorbs onto the active carbon sites of the resin pyrolytic product, forming chemically adsorbed oxygen; (2) The chemically adsorbed oxygen on the active carbon sites of the resin carbon reacts with the active carbon to form a transition state of carbon monoxide; (3) The transition state of carbon monoxide desorbs from the surface of the resin carbon. The apparent oxidation kinetic parameters were calculated from the mass-loss data obtained during the main oxidation stage. The oxidation rate was described using the Arrhenius Equation (1):

(1) $${K\;=\;A_{a}e}^{\frac{-E_{a}}{\mathrm{RT}}}$$

where K is the oxidation reaction rate, Aa is the apparent frequency factor, Ea is the apparent oxidation activation energy, R is the gas constant, and T is the absolute temperature. In the present non-isothermal TG experiment, W is defined as the fractional mass loss during oxidation, and the heating rate is defined as β = dT/dt. Therefore, the oxidation rate can be expressed as K = dW/dt = β(dW/dT). By applying a logarithmic transformation to the Arrhenius equation, the oxidation kinetic equation of PRPC was derived as in Equation (2):

(2) $$\mathrm{Ln}\left(\beta \frac{\mathrm{dW}}{\mathrm{dT}}\right)\;=\;\ln\left(A_{a}\right)\;-\;\frac{E_{a}}{R}\;\times \;\frac{1}{T}$$

Accordingly, Ea and Aa were obtained from the slope and intercept of the ln K versus 1/T plots, respectively. It should be noted that the calculated Ea is an apparent activation energy obtained under non-isothermal conditions within the selected oxidation region, rather than an intrinsic constant describing the entire oxidation process. This treatment provides a comparative kinetic parameter for the selected oxidation region rather than a complete description of the entire oxidation process.

For X-ray diffraction (XRD) phase analysis, PRPC was ground to a particle size of ≤0.045 mm and examined using an X’Pert MPD PRO diffractometer (Philips, Eindhoven, The Netherlands) with Cu K_α1_ radiation operated at 40 mA and 40 kV. The diffraction patterns were collected over a 2θ range of 10°–90° at a scanning rate of 2°/min.

The crystallite sizes of Cu and Cu_2_O in the thermal decomposition products of the copper salts were calculated using the Debye–Scherrer Formula (3).

(3) $$D=\frac{K\lambda }{\beta \cos\theta }$$

In this equation, λ denotes the wavelength of the incident X-rays, β corresponds to the full width at half maximum (FWHM) of the diffraction peak, and θ represents the diffraction angle.

The PRPC obtained under different conditions was ground into fine powder (≤0.045 mm) and characterized using a laser confocal micro-Raman spectrometer (Raman, inVia Qontor, Renishaw, Wotton-under-Edge, UK) to evaluate changes in the graphitic structure of the carbonaceous components. The microstructural morphology of PRPC was examined using a field-emission scanning electron microscope (FESEM, Nova NanoSEM 400, FEI Company, Philips, Hillsboro, OR, USA) operated at an accelerating voltage of 15–30 kV. The elemental composition of the samples was determined by energy-dispersive X-ray spectroscopy (EDS; INCA IE 350 Penta FET X-3, Oxford, Oxfordshire, UK) using a Si(Li) standard detector with an energy resolution of less than 129 eV, an elemental detection range from C (Z = 6) to U (Z = 92), and a peak-to-background ratio exceeding 2000:1.

The microstructure of PRPC was characterized by high-resolution transmission electron microscopy (HR-TEM; JEM-2100UHR STEM/EDS, JEOL, Tokyo, Japan) at an accelerating voltage of 200 kV. The chemical composition of microregions was detected using an energy-dispersive X-ray spectrometer (EDS, Noran623M-3SUT, Thermo Electron Corporation, Waltham, MA, USA) equipped with a Si(Li) standard detector with an energy resolution better than 133 eV and an elemental detection range from boron (Z = 5) to uranium (Z = 92).

## 3. Results and Discussion

### 3.1. Evolution Process of Catalyst Precursors

**Figure 1:**
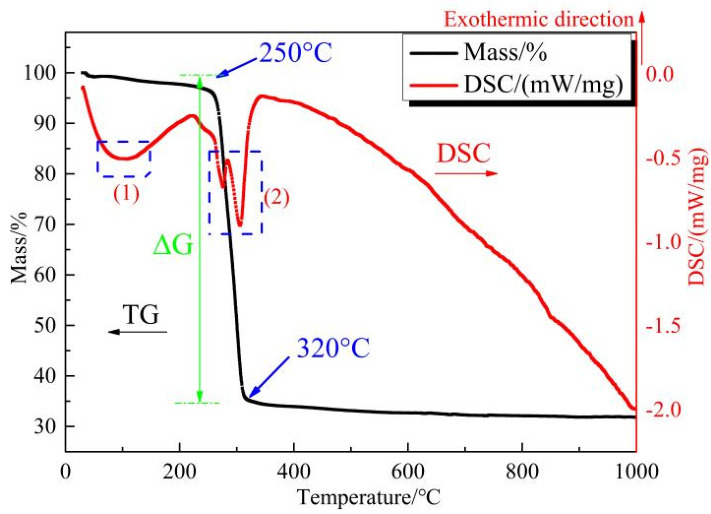
TG-DSC curves of cupric tartrate.

To evaluate the thermal decomposition behavior of the catalyst precursor, cupric tartrate was heated under an argon atmosphere from 30 °C to 1000 °C at a heating rate of 10 °C/min. The thermal decomposition behavior of cupric tartrate was first characterized by TG-DSC, and the results are presented in Figure 1. The TG curve in Figure 1 indicated that cupric tartrate decomposition initiated at 250 °C and ended at 320 °C, with a mass loss of approximately 65%. The corresponding DSC curve shows two endothermic peaks. The first peak (60–140 °C) corresponded to dehydration, involving the loss of both crystalline and coordinated water. The second peak (250–320 °C) arose from decarboxylation and decomposition of the organic moiety. The TG-DSC results indicated that the thermal decomposition products of cupric tartrate exhibited excellent stability, suggesting their potential relevance for subsequent catalytic applications.

**Figure 2:**
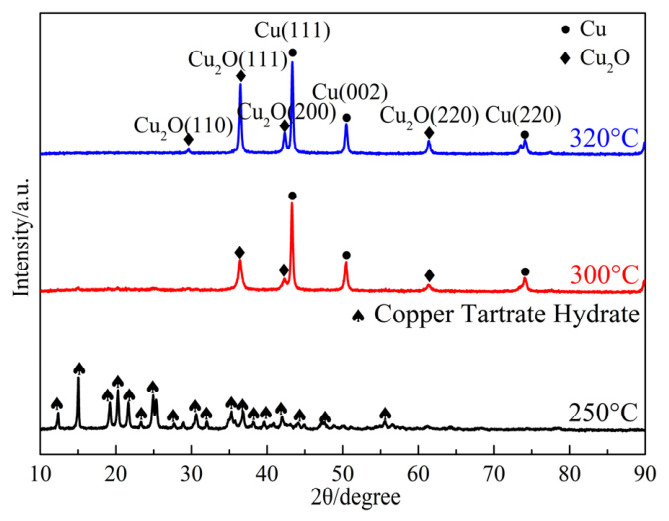
XRD patterns of cupric tartrate heated at 250 °C, 300 °C and 320 °C.

To further investigate the phase composition of cupric tartrate pyrolysis products, the cupric tartrate was heated separately to 250 °C, 300 °C and 320 °C at 10 °C/min in flowing argon and held for 1 h at each temperature. The resulting XRD patterns were obtained, as shown in Figure 2.

The XRD pattern of cupric tartrate heated at 250 °C exhibited predominant diffraction peaks assignable to cupric tartrate hydrate (ICDD 00-001-0158), indicating that cupric tartrate had not decomposed and confirming its thermal stability at this temperature. The XRD pattern of cupric tartrate heated at 300 °C revealed characteristic diffraction peaks corresponding to Cu (ICDD 96-710-1270) and Cu_2_O (ICDD 01-078-2076), demonstrating partial decomposition into these phases. As the heat treatment temperature increased to 320 °C, the diffraction peaks of Cu and Cu_2_O intensified significantly, indicating progressive conversion to stable Cu/Cu_2_O phases. Based on Debye–Scherrer calculations, the crystallite sizes of Cu and Cu_2_O were 35.9 nm and 16.4 nm, respectively, in the 300 °C decomposition product and increased to 39.7 nm and 33.6 nm at 320 °C. Collectively, the XRD analysis confirmed that Cu and Cu_2_O are the primary decomposition products of cupric tartrate and that their crystallite sizes increase with temperature. This microstructural evolution likely governs variations in catalytic performance.

**Figure 3:**
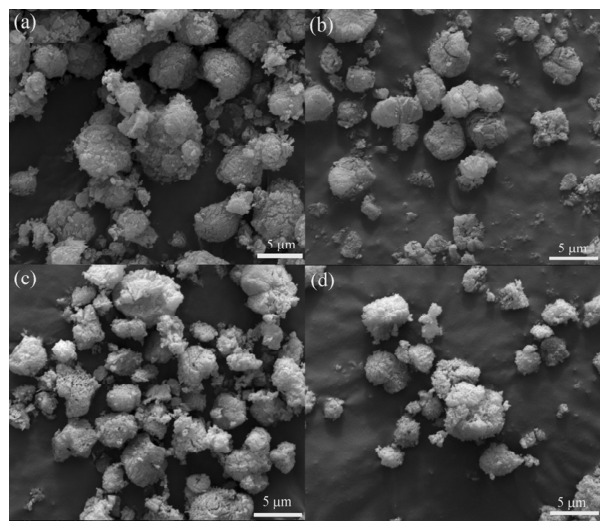
SEM images of cupric tartrate heated at different temperatures, (**a**) initial; (**b**) 250 °C; (**c**) 300 °C and (**d**) 320 °C.

To further investigate the microstructure of cupric tartrate pyrolysis products, the SEM images were obtained, as shown in Figure 3.

Figure 3a shows that the initial cupric tartrate consisted of granular aggregates with diameters of 5–10 μm. After heating at 250 °C (Figure 3b), the morphology remained largely unchanged, exhibiting negligible deviation from that of the precursor. This microstructural stability corroborates the absence of significant thermal decomposition at 250 °C, consistent with XRD phase analysis. The voids observed on particle surfaces after calcination at 300 °C (Figure 3c) are attributable to carboxyl bond cleavage in cupric tartrate and the subsequent release of volatile organic fragments. When the calcination temperature was increased to 320 °C (Figure 3d), intensified pore development on the particle surfaces accompanied the decomposition of cupric tartrate into nanoscale Cu/Cu_2_O mixtures.

These findings demonstrate that decomposition of cupric tartrate initiates at 250 °C, yielding thermally stable nanoscale Cu/Cu_2_O composites beyond 320 °C. Given that Cu nanoparticles have been reported to be active for the catalytic decomposition of hydrocarbon gases [21,22,23,24,25], the Cu/Cu_2_O composites derived from cupric tartrate are expected to exhibit catalytic activity toward carbon nanostructure formation during phenolic resin pyrolysis. This hypothesis is further examined in Section 3.2 through microscopic observations of the pyrolyzed products.

### 3.2. Effect of Cupric Tartrate on Phenolic Resin

**Figure 4:**
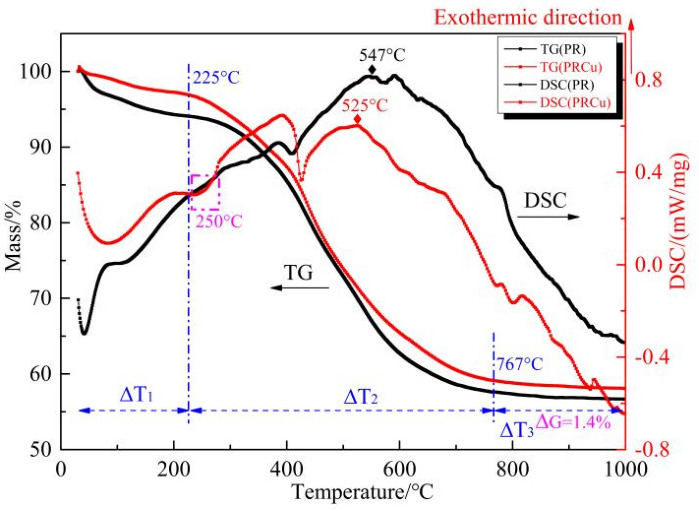
TG-DSC curves of PR and PRCu.

To determine the influence of introducing the cupric tartrate catalyst precursor on the thermal decomposition behavior of phenolic resin, PR and PRCu samples were heated in an argon atmosphere at a rate of 10 °C/min from 30 °C to 1000 °C, and the resulting TG-DSC curves were obtained, as shown in Figure 4.

Figure 4 compares the TG-DSC profiles of PR and PRCu recorded during heating from 30 to 1000 °C under argon at 10 °C/min, thereby illustrating the effect of the catalyst precursor on the thermal decomposition behavior of phenolic resin. The TG-DSC curves indicate that the thermal decomposition-related mass loss of PR occurred predominantly in three stages. The first-stage (30–225 °C) proceeded gradually, with a mass reduction of approximately 6%, which was mainly attributed to the physical desorption of water and the release of low-molecular-weight compounds. The second-stage (225–767 °C) showed rapid mass loss, with a steep 38% reduction in the TG curve, resulting from scission of methylene bridge bonds (-CH_2_-) and ether bonds (-CH_2_-O-CH_2_-) in phenolic resin and the release of substantial gaseous products (H_2_, CH_4_, CO, CO_2_). Above 767 °C, the mass-loss rate became extremely low and stable because gas evolution was drastically reduced during deep dehydrogenation, aromatization, and condensation reactions, with hydrogen (H_2_) as the dominant gaseous product and minor carbon monoxide (CO) also released. The DSC curve of PRCu exhibits an endothermic peak at 250 °C, corresponding to decomposition of the cupric tartrate precursor. The exothermic peak of PRCu shifted from 547 °C to 517 °C, which may be attributed to Cu particles derived from cupric tartrate decomposition. These particles may facilitate low-temperature carbon nanostructure formation during phenolic resin pyrolysis, thereby accelerating heat release. Compared with pure phenolic resin, PRCu shows a smoother mass-loss profile in the TG analysis. The 1.49% reduction in total mass loss confirms that the catalyst precursor delays thermal decomposition and enhances the char yield.

**Figure 5:**
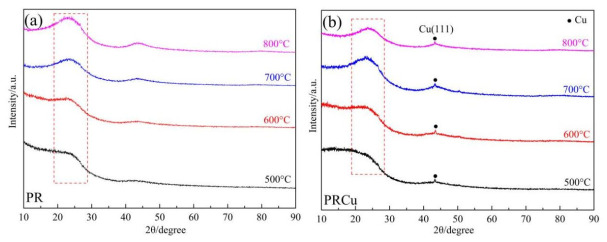
XRD patterns of PR (**a**) and PRCu (**b**) calcined at 500–800 °C.

To further investigate the influence of cupric tartrate on the phase composition of PRPC, Figure 5 presents the XRD patterns of PR and PRCu calcination at 500–800 °C. The XRD pattern of PR showed no distinct diffraction peaks. In contrast, phenolic resin containing cupric tartrate exhibited a strong diffraction peak at 43.3°, corresponding to the Cu(111) crystal plane of metallic Cu (ICDD 96-710-1270), with no detectable Cu_2_O signals. This confirms the complete reduction of Cu_2_O to metallic Cu by the reducing gases generated during resin pyrolysis. Remarkably, Cu remained stable in its metallic form within 500–800 °C, suggesting high catalytic potential for carbon nanostructure formation.

**Figure 6:**
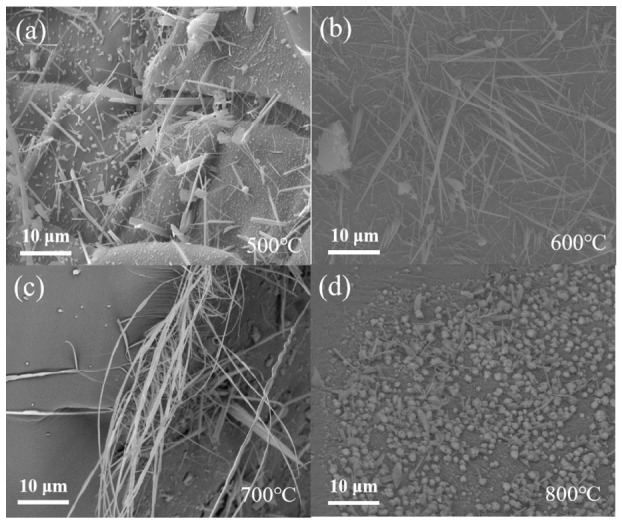
SEM images of PRCu calcined at 500–800 °C: (**a**) 500 °C, (**b**) 600 °C, (**c**) 700 °C and (**d**) 800 °C.

Figure 6 presents SEM images of PRCu pyrolyzed at 500–800 °C, further clarifying the influence of metallic Cu on the microstructure of phenolic resin-derived carbonized products. At 500 °C, carbon nanofibers (CNFs) formed on the PRCu product (Figure 6a), with lengths of 10–20 μm. Increasing temperature to 600 °C enhanced CNF yield and extended the fiber length to 20–30 μm (Figure 6b), which was attributed to the increased release of phenolic resin-derived gases that provided abundant carbon sources for Cu-catalyzed growth, while the higher thermal energy further promoted fiber elongation. At 700 °C, despite a reduced CNF quantity caused by the declining availability of gaseous carbon precursors, the maximum fiber length reached 30–50 μm (Figure 6c) through thermally accelerated growth. After pyrolysis at 800 °C, sparse CNFs shorter than 10 μm coexisted with coarsened Cu particles (Figure 6d), resulting from insufficient carbon supply and temperature-induced particle aggregation, which reduced the catalytic efficiency.

**Figure 7:**
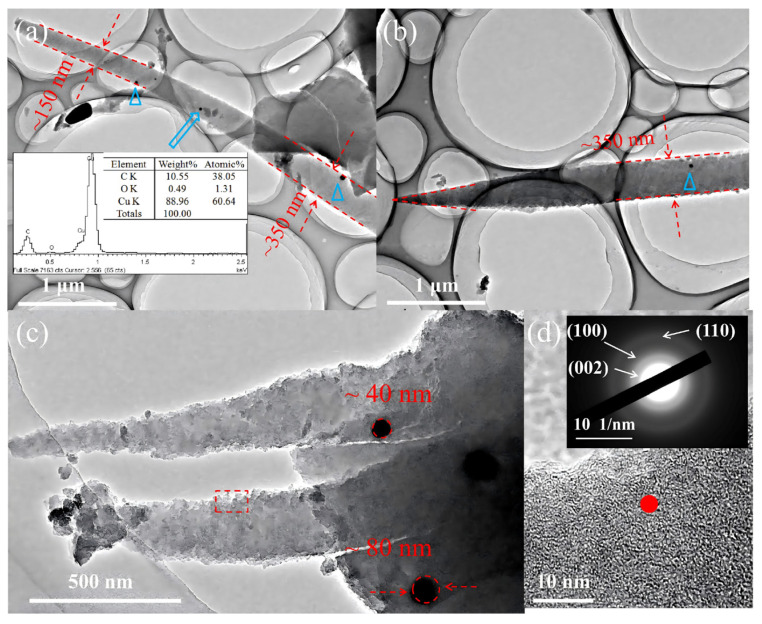
TEM images of samples PRCu calcined at 500 °C for 3 h. (**a**) The bottom of carbon nanofiber (The spectrum corresponds to the blue arrow in the diagram); (**b**)The tip of carbon nanofibers; (**c**) Carbon nanofibers containing catalyst particles; (**d**) The HRTEM image and SAED of the rectangular area in (**c**).

Figure 7 presents TEM/HR-TEM images of Cu-catalyzed carbon nanofibers fabricated from PRCu at 500 °C, further clarifying their morphology.

Figure 7a shows smooth-surfaced carbon nanofibers with diameters tapering from approximately 350 nm in the middle section to approximately 150 nm at the tip. EDS analysis of the embedded nanoparticles showed dominant C (38.05 at%) and Cu (60.64 at%), confirming their metallic Cu nature despite the presence of 1.31 at% residual oxygen. This minimal oxygen content is attributed to incomplete reduction of Cu_2_O derived from cupric tartrate decomposition. Figure 7b reveals a carbon nanofiber with a middle-section diameter of approximately 350 nm that tapers into a needle-like tip. This conical morphology likely results from progressively diminishing carbon concentration gradients during catalytic elongation. The tapered carbon nanofibers in Figure 7c contain basal metallic Cu nanoparticles (40–80 nm) with polydispersity and irregular contours. HR-TEM analysis of the framed region was used to quantify the local structural ordering level. HR-TEM and SAED analysis of the framed region in Figure 7d reveals low-crystallinity or turbostratic carbon, as indicated by disordered lattice features and concentric diffraction rings corresponding to graphite-type carbon planes. These observations indicate that the CNFs are turbostratic in nature, with nanoscale ordered domains, rather than being highly crystalline graphite.

**Figure 8:**
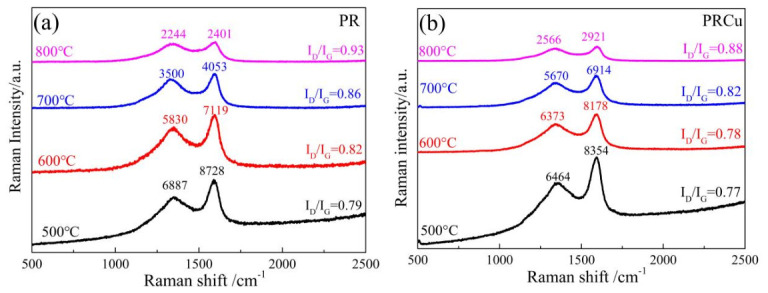
Raman patterns of PR (**a**) and PRCu (**b**) calcined at 500–800 °C.

Raman spectroscopy was employed to evaluate the influence of Cu-derived species on the local carbon structure of phenolic resin-derived pyrolytic carbon. The D band at approximately 1340 cm^−1^ is generally associated with defects and breathing modes of sp^2^ carbon rings, whereas the G band near 1600 cm^−1^ originates from in-plane stretching vibration of sp^2^-hybridized carbon. However, for disordered and polymer-derived carbons, the intensity ratio I_D_/I_G_ should not be interpreted as a simple monotonic indicator of crystalline graphitization. Instead, according to established Raman models for disordered carbon, variations in I_D_/I_G_ reflect changes in sp^2^ cluster size, defect density, aromatic domain evolution, and short-range structural ordering [27]. The Raman spectra of PR (a) and PRCu (b) calcined at 500–800 °C are shown in Figure 8.

Figure 8 shows that the R values of pure phenolic resin (PR) pyrolyzed at 500 °C, 600 °C, 700 °C, and 800 °C for 3 h were 0.79, 0.82, 0.86, and 0.93, respectively.

The PRCu samples exhibited lower ID/IG values than the corresponding PR samples, suggesting that cupric tartrate-derived Cu species promoted local sp^2^ structural rearrangement and short-range ordering in the carbon matrix. Nevertheless, the HR-TEM and SAED results showed disordered lattice features and concentric diffraction rings, indicating that the carbon nanofiber-like structures were low-crystallinity or turbostratic carbon rather than highly crystalline graphite. Therefore, the structural evolution induced by Cu species is described here as enhanced local sp^2^ ordering or turbostratic ordering, rather than complete graphitization.

**Figure 9:**
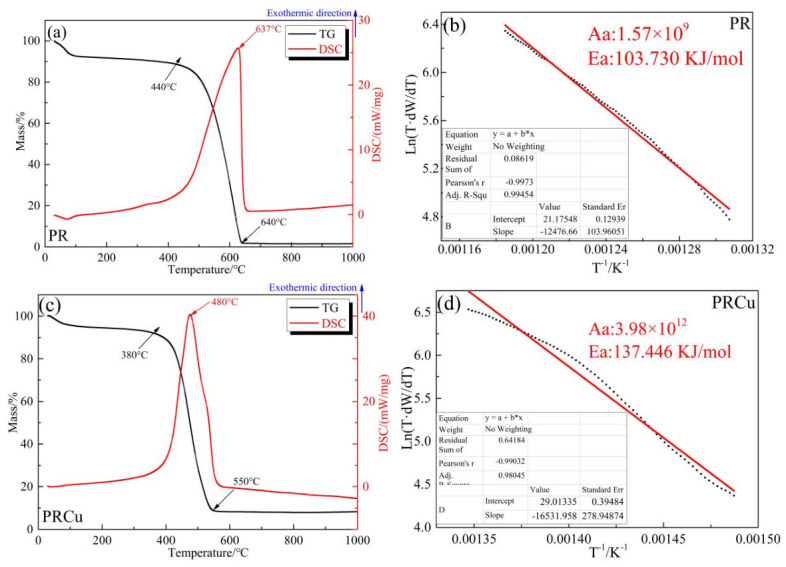
TG–DSC curves and the oxidation reaction kinetics curves of samples PR and PRCu. (**a**) TG–DSC curves of sample PR; (**b**) The oxidation reaction kinetics curves of sample PR; (**c**) TG–DSC curves of PR; (**d**) The oxidation reaction kinetics curves of sample PRCu.

Cu-catalyzed nanofibers may simultaneously enhance structural ordering and fill pores in phenolic resin-derived carbons, thereby potentially improving oxidation resistance. The oxidation kinetics of PR and PRCu chars pyrolyzed at 800 °C were investigated by TG-DSC in air. Regression analysis of the mass-loss profiles yielded oxidation kinetic parameters, and the calculated apparent activation energies (Ea) and frequency factors (Aa) for each sample are shown in Figure 9.

TG-DSC analysis (Figure 9a) reveals that PRPC initiated oxidation at 440 °C and underwent complete combustion by 640 °C, exhibiting a sharp exothermic peak at 637 °C. Figure 9b shows that the apparent oxidation activation energy, Ea, of PR pyrolytic carbon was calculated to be 103.730 kJ/mol. Figure 9c shows that PRCu pyrolytic carbon began to oxidize at 380 °C and was completely oxidized by 550 °C, with the exothermic peak shifted to 480 °C. Figure 9d shows that the apparent oxidation activation energy, Ea, of PRCu pyrolytic carbon was calculated to be 137.446 kJ/mol.

The oxidation initiation temperature is an apparent parameter, while the activation energy (Ea) is an intrinsic kinetic parameter, reflecting the sensitivity of the reaction rate to temperature rather than the absolute temperature at which oxidation occurs. Figure 9a,c shows that PRCu starts to oxidize at a lower temperature and reaches complete oxidation earlier than PR, this behavior may be related to the possible catalytic effect of residual Cu/CuO species on oxygen activation and carbon oxidation [28]. When elemental Cu is heated in the air, it will oxidize and turn into CuO. CuO acts as an electron acceptor. The phenolic resin carbonization product transfers electrons to CuO, losing one π bond and weakening the C-C bonds in the carbon structure of the carbonized product. This promotes the evolution process of CO and accelerates the oxidation process of the carbonized product.

Figure 9d shows that the apparent oxidation activation energy, Ea, of PRCu pyrolytic carbon was calculated to be 137.45 kJ/mol. At first glance, this higher apparent activation energy appears to contrast with the lower onset and burnout temperatures observed for PRCu. However, the apparent activation energy obtained from the kinetic fitting reflects the fitted energy barrier within the selected mass-loss/conversion region—specifically, the main oxidation stage—and should not be used alone as definitive proof of overall oxidation-resistance improvement. Rather, the oxidation behavior of PRCu should be understood as a combined effect: the lower onset and burnout temperatures suggest that the material is more readily oxidized at low temperatures due to the catalytic influence of Cu species and the surface characteristics of the CNFs, whereas the higher apparent activation energy indicates that, once the oxidation reaction enters the main stage, the structurally more ordered carbon matrix presents a higher kinetic barrier against further oxidation.

In summary, the addition of cupric tartrate does not universally improve the oxidation resistance of the pyrolytic carbon across the entire temperature range; instead, it introduces a dual effect-promoting low-temperature oxidation reactivity while increasing the activation energy for the main oxidation stage.

### 3.3. Proposed Formation Process of Carbon Nanofiber-like Structures

**Figure 10:**
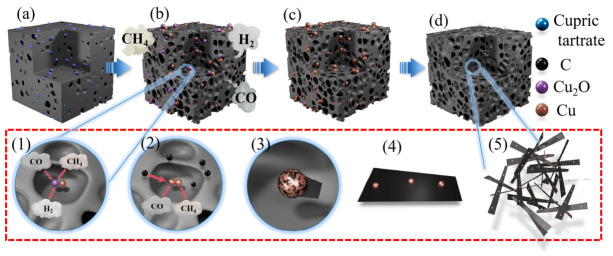
The proposed formation process of carbon nanofiber-like structures in the PRCu system. (**a**) curing process of phenolic resin; (**b**) pyrolysis process of phenolic resin; (**c**) the reduction process of nanocopper particles; (**d**) the dissolution−precipitation process of carbon nanofiber. (The image (1)–(5) in figure indicates the progression of the growth process of carbon nanofibers catalyzed by nano-copper).

Figure 10 schematically illustrates the proposed formation process of carbon nanofiber-like structures in the PRCu system. The following discussion is based on the experimental observations (SEM, TEM, XRD, Raman) combined with well-established literature reports [20,21,22,23,24,25,26], and should be regarded as a reasonable hypothesis rather than a directly proven mechanism.

During sample curing, phenolic resin forms ether bonds through the dehydration condensation of phenolic hydroxyl groups, thereby releasing small-molecule gases. Meanwhile, alcohol also evaporates during this stage. The combined effect of these processes produces a well-developed pore structure in the cured sample. Cupric tartrate undergoes thermal decomposition to form Cu/Cu_2_O during this stage, as shown in Figure 10a.

As the heat treatment temperature increases, the pyrolysis of phenolic resin involves the cleavage of methylene and ether bonds, producing reducing gases such as CH_4_, CO and H_2_ that accumulate in the pores formed during curing, as shown in Figure 10b. With continued thermal decomposition, Cu_2_O may be reduced to metallic Cu by these reducing gases through the following reactions (4), (5), and (6), as schematically shown in Figure 10(1):

4Cu_2_O(s) + CH_4_(g) → 8Cu(s) + CO_2_(g) + 2H_2_O(g) (4)

Cu_2_O(s) + CO(g) → 2Cu(s) + CO_2_(g) (5)

Cu_2_O(s) + H_2_(g) → 2Cu(s) + H_2_O(g) (6)

The atomic states at the surface and in the interior of nano-Cu differ. The interior has a relatively well-crystallized structure, whereas the surface atoms are less coordinated and more unstable. To minimize the system energy, the surface atoms undergo relaxation and structural rearrangement, resulting in a melting point for nano-Cu that is lower than that of bulk copper. Zhang et al. [21] found that the melting point of nano-Cu decreases as particle size decreases. When the nano-Cu particle size is approximately 50 nm, its melting point is 470 °C. Therefore, the Cu particles formed in this work may exhibit high surface activity at the pyrolysis temperatures employed.

At a pyrolysis temperature of 500 °C, Cu nanoparticles may become highly active and may partly evolve into droplet-like forms. Under these conditions CO, CH_4_, and other carbon-containing gases generated during phenolic resin pyrolysis may adsorb on the Cu surface and undergo catalytic decomposition to form reactive carbon species [29]. As shown in Figure 10(2), due to the very limited solubility of carbon in Cu nanoparticles, the growth process should be considered as a Cu-assisted surface-mediated precipitation process or a limited dissolution–precipitation-like process, rather than a classical bulk dissolution–precipitation mechanism [30]. The observed tapered morphology is consistent with this proposed process, in which carbon species generated near the Cu-rich particles progressively deposit and extend into fiber-like structures [31]. Differences in exposed Cu facets, particle size, particle geometry, and local carbon supply may lead to anisotropic growth and the formation of tapered or conical morphologies [32]. As the reaction proceeds, these fiber-like structures may stack into cluster-like networks that cover parts of the resin-derived carbon matrix and partially fill pores, as shown in Figure 10(5).

## 4. Conclusions

In this study, cupric tartrate was used as a copper precursor to modify the microstructural evolution and oxidation behavior of phenolic resin-derived carbon. The main conclusions are as follows:

(1) Cupric tartrate was found to undergo thermal decomposition at 250–320 °C into nanoscale Cu/Cu_2_O composites under argon, and subsequent reduction to metallic Cu nanoparticles occurs under the reducing atmosphere generated by phenolic resin pyrolysis. These findings demonstrate that cupric tartrate can act as an effective in situ precursor for Cu species in phenolic resin pyrolytic carbon.
(2) The in situ formed Cu nanoparticles were observed to be associated with the growth of tapered carbon nanofibers, with the maximum fiber length reaching 30–50 μm at 700 °C. On the basis of these experimental observations and previously reported studies on Cu-catalyzed carbon deposition, a dissolution–precipitation pathway is proposed as a plausible mechanism for CNF formation.
(3) The presence of Cu-catalyzed CNFs correlates with a decreased Raman I_D_/I_G_ ratio, indicating enhanced structural ordering of the pyrolytic carbon. Combined with HR-TEM and SAED observations, this result indicates that Cu species promoted local sp^2^ hybridization and turbostratic ordering in the carbon matrix, rather than forming highly crystalline graphite.
(4) The oxidation behavior of phenolic resin pyrolytic carbon was significantly altered by the addition of cupric tartrate, with an increased apparent oxidation activation energy from 103.73 to 137.45 kJ/mol. However, the onset oxidation temperature of PRCu was observed to be lower than that of PR, which is attributed to the catalytic effect of residual Cu species on the oxidation of phenolic resin pyrolytic carbon.

In summary, this work provides a facile strategy for the in situ catalytic modification of phenolic resin-derived carbon using cupric tartrate as a catalyst precursor, and offers a framework for understanding the structural evolution and oxidation behavior of the resulting carbon materials.

## Data Availability

The original contributions presented in this study are included in this article. Further inquiries can be directed to the corresponding authors.
